# Comparative Proteomic Analysis of the Effect of Periplocoside P from *Periploca sepium* on Brush Border Membrane Vesicles in Midgut Epithelium of *Mythimna separata* Larvae

**DOI:** 10.3390/toxins10010007

**Published:** 2017-12-22

**Authors:** Mingxing Feng, Yankai Li, Xueting Chen, Quansheng Wei, Wenjun Wu, Zhaonong Hu

**Affiliations:** 1 Institute of Pesticide Science, College of Plant Protection, Northwest A&F University, Yangling, Shaanxi 712100, China; fengmx2010@126.com (M.F.); liyankai1991@163.com (Y.L.); wacxt@163.com (X.C.); woshiweiquansheng@163.com (Q.W.); wuwenjun@nwsuaf.edu.cn (W.W.); 2 Provincial Key Laboratory for Botanical Pesticide R&D of Shaanxi, Yangling, Shaanxi 712100, China; 3 Key Laboratory of Crop Pest Integrated Management on the Loess Plateau, Ministry of Agriculture, Yangling, Shaanxi 712100, China

**Keywords:** botanical pesticide, periplocoside P, proteomics, brush border membrane vesicles (BBMVs), V-type ATPase A subunit

## Abstract

Periplocoside P (PSP), a novel compound isolated from *Periploca sepium* Bunge, possesses insecticidal activity against some lepidopterans, such as *Mythimna separata*. In *M. separata,* the brush border membrane vesicles of the midgut epithelium are the initial site of action of periplocosides. We conducted two-dimensional gel electrophoresis and matrix-assisted laser desorption/ionization time of flight/time of flight mass spectrometry analysis to analyze differentially expressed proteins (DEPs) from periplocoside P (PSP)-treated *M. separata*. We successfully isolated seven up-regulated and three down-regulated DEPs that have been previously identified, as well as a novel DEP. The DEPs are implicated in protein degradation, transporter, folding, and synthesis, and in juvenile hormone biosynthesis. DEPs involved in the oxidative phosphorylation energy metabolism pathway are enriched. Through real-time polymerase chain reaction assay, we confirmed that *vma1* expression is significantly up-regulated expression levels in PSP-treated *M. separata* larvae. Enzymology validation further indicated that PSP can significantly inhibit V-type ATPase activity in a concentration-dependent manner. Given these results, we speculate that in *M. separata*, the V-type ATPase A subunit in the midgut epithelium is the putative target binding site of periplocosides. This finding provides preliminary evidence for the mode of action of periplocosides.

## 1. Introduction

Target-oriented design has become the modern mainstream model for the development of novel pesticides [1]. The identification and validation of novel targets provide crucial guidance to the development of novel pesticide with special mechanisms of action [2]. Moreover, proteomics technology has expanded the research and development of pesticide targets [3].

*Periploca sepium* Bunge, a member of the *Asclepiadaceae* family [4], has extensive applications in the treatment of human diseases [5,6], as well as excellent insecticidal action against several lepidopteran pests [7,8,9]. Our research group has focused on the isolation and identification of systemically active compounds from *P. sepium* [10,11,12]. Periplocoside P (PSP), a novel compound isolated from *P. sepium* has high insecticidal activity (LC_50_ = 110 mg·L^−1^) against *Mythimna separata* [13]. To determine the specific mechanism of action of periplocosides against *M. separata* larvae, we performed systematic research and showed that putative targets of periplocosides may exist in the brush border membrane vesicles (BBMVs) of the larvae midgut [13,14]. Nevertheless, the specific site of action of periplocoside compounds remains unknown.

Proteomics has been utilized to identify and verify insecticide targets in many insects. Candas et al. found that midgut chymotrypsin-like proteinase is directly responsible for *Bacillus thuringiensis* (*Bt*) resistance in Indian mealmoth (*Plodia interpunctella*) [15]. Cry1Ac binds to actin and alkaline phosphatase (ALP) in tobacco hawkmoth (*Manduca sexta*) [16]. Five binding proteins are responsible for the resistance of the diamondback moth (*Plutella xylostella*) against Cry1Ac [17]. Additionally, four Cry1Ac toxin targets may exist in the bollworm (*Helicoverpa armigera*) midgut [18]. The application of different proteomic approaches will improve our understanding of the functional mechanism of pesticides.

In this study, we utilized two-dimensional gel electrophoresis (2-DE) and matrix-assisted laser desorption/ionization time of flight/time of flight mass spectrometry (MALDI-TOF/TOF MS) to investigate the putative target proteins of PSP in the midgut BBMVs of *M. separata* larvae. Our present findings have an important role in our future studies on the mode of action of periplocosides against crop insect pests.

## 2. Materials and Methods

### 2.1. Compounds

**Figure 1:**
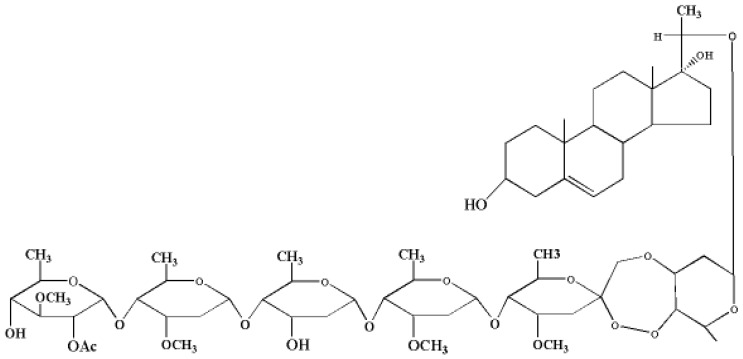
Chemical structure of periplocoside P.

PSP was extracted from the root bark of *Periploca sepium* Bunge and purified in our laboratory [19]. The specific molecular structure of PSP is presented in Figure 1. High-performance liquid chromatography monitoring indicated that the purity of PSP used in this study exceeded 98%.

### 2.2. Insect Rearing and Treatment

Fifth-instar larvae of *M. separata* were provided by our laboratory. The larvae were derived from a susceptible strain that has been cultured for 20 years under the laboratory conditions of 25 °C, 70% relative humidity, and a 16 h/8 h light/dark cycle. *M. separata* larvae that had newly entered fifth-instar were starved for 24 h to ensure that the food was digested and excreted at the maximum extent. Then the *M. separata* larvae that maintained full vitality and normal metabolism were selected for the following experiments. Wheat leaves (0.5 cm × 0.5 cm) were coated with 1 μL of 4 µmol/L PSP solution (dilulted with acetone) or acetone alone, and then fed to selected *M. separata* larvae.

### 2.3. BBMVs Preparation

The midguts of fifth-instar *M. separata* larvae were collected [20]. The peritropic maroundrix and food residues were discarded. The remaining midgut tissue was washed with 0.7% NaCl and dehydrated with filter paper. BBMVs were extracted through the differential centrifugation method [21]. Briefly, Midguts (500 mg) were homogenized in a nine-fold volume of buffer solution (300 mmol/L mannitol, 5 mmol/L ethylene glycol bis(α-aminoethyl ether)-*N*,*N*′-tetracetic acid (EGTA), 17 mmol/L *tris*-(hydroxymethyl) aminomethane (Tris)-HCl, pH 7.5) containing a protease inhibitor cocktail (Complete, Roche, Indianapolis, IN, USA). An equal volume of 24 mmol/L MgCl_2_ were added to homogenized tissue and incubated on ice for 15 min. Then the mixture was centrifuged at 2500 *g* for 15 min at 4 °C. The supernatant was saved on ice, and the pellet was suspended in half original volume of above buffer solution containing a protease inhibitor cocktail. The resuspension was centrifuged at 2500 *g* for 15 min again, and the twice suspension was pooled and centrifuged at 30,000 *g* for 30 min at 4 °C. The supernatant was discarded, and the BBMV pellet was resuspended in 100 µL ice-cold above buffer solution containing a protease inhibitor cocktail. Protein concentrations were determined with a 2-DE Quant Kit (GE Biosciences, Piscataway, NJ, USA). Proteins were stored at −80 °C until further use.

### 2.4. 2-DE

BBMV proteins were purified with the 2D Clean-Up Kit (GE Biosciences, Piscataway, NJ, USA) in according with Viswanathan’s method [22]. Isoelectrofocusing proceeded by linearizing 18 cm IPG strips (pH 3–10) containing 300 µg of protein samples on IPGphor instrument (GE Biosciences) at 60 KV/h. Focused strips were first equilibrated in 2% DTT (*w*/*v*) and then in 2.5% iodoacetamide (*w*/*v*) in equilibration buffer. Each equilibration step was performed for 15 min. Sodium dodecyl sulfate-polyacrylamide gel electrophoresis (SDS-PAGE) was performed with 12% gel using Protein II device. Gels obtained from SDS-PAGE were dyed with Coomassie Blue G250. Each treatment was performed with at least three biological replicates.

### 2.5. Image Acquisition and Data Analysis

After discoloration, images were scanned with a GS710 scanner and trimmed, optimized, and analyzed using PDQuest Version 8.0 software. The image with the maximum number of points and minimal streaks was selected as the standard image, and protein spots were automatically detected. After automatic detection, some spots were still undetected or “false points”, which should be added, deleted or segmented by hand. Undetected spots were added to or removed from the standard map in accordance with the consistency of repeated groups. All protein spots were quantified by using PDQuest software. The differential expression levels of proteins were confirmed through qualitative (>1.5-fold) and Student’s *t*-tests (*p* < 0.05). Each treatment was performed with at least three replicates.

### 2.6. Protein Identification and Bioinformatics Analysis

Proteins bands were excised from gels with an Eppendorf pipette and repeatedly washed with ddH_2_O. Proteins were digested through the in-gel digestion method [23]. Extracted peptides were dissolved in a CHCA-saturated solution. MALDI-TOF/TOF MS analysis was outsourced to the Beijing Genomics Institute (Beijing, China). The MS data of the peptides were imported into the MASCOT Sequence Query server for comparative analysis with a confidence interval of 95%. Results with a probability score value exceeding 90 were considered as successful protein identification. For bioinformatics analysis, the related functional information of differential expressed proteins (DEPs) was obtained though the UniProt database (http://www.uniprot.org). Hierarchical clustering analysis was performed with Genesis 1.7.6 software. Gene ontology (GO) was performed using STRING (version 10.0), which is based on the Kyoto Encyclopedia of Genes and Genomes database. This experiment was performed with at least three biological replicates.

### 2.7. Quantitative Determination of Gene Expression Levels

Total RNA was extracted from the midguts of *M. separata* larvae using Trizol Reagent kit (TaKaRa, Dalian, China). RNA concentration was quantified using an ultraviolet spectrophotometer (ACTGene, Piscataway, NJ, USA). First-strand copy DNA (cDNA) was synthesized with PrimeScript™ RT Reagent Kit (TaKaRa). Real-time quantitative polymerase chain reaction (RT-qPCR) was conducted with a SYBR Premix Ex TaqII (TaKaRa). *β-actin* (GQ856238) was selected as the endogenous control, and the related primers are shown in Table 1. RT-qPCR was conducted with 20 μL of reaction mixture containing 10 μL of SYBR buffer, 1.0 μL of cDNA, 1.0 μL of primers, and 8.0 μL of ddH_2_O. PCR was conducted with 35 cycles under the following conditions: 95 °C for 30 s, 95 °C for 10 s, and 72 °C for 15 s. An ultimate extension stage was conducted at 72 °C for 10 min. The relative amounts of PCR products were calculated through the 2^−ΔΔCt^ method.

### 2.8. Determination of Enzyme Activity

Aminopeptidase N (APN) activity was measured on the basis of Hafkenscheidat’s method [24]. PSP solutions were prepared with dimethyl sulfoxide (DMSO) at the final concentrations of 20, 40, 80, and 200 µmol/L. Bestatin (10 µmol/L) was set as the positive control. ALP activity was examined in line with the method used by Lowry et al. [25]. Specific activity was expressed in units per milligram protein. V-ATPase activity was determined in accordance with the method of Tiburcy et al. [26]. The concentration of PSP was set to that of aminopeptidase. Bafilomycin A1 (BA1, 3 µmol/L, 10 µL) was set as the positive control. Inorganic phosphate was measured through the method of Wieczorek et al. [27]. All enzyme activity tests mentioned above were performed with at least three biological replicates per treatment.

### 2.9. Data Statistics Analysis

Data was analyzed with SPSS 20.0 software (SPSS, Chicago, IL, USA). Data were represented as mean ± SD form. Analysis of variance (ANOVA) was performed to identify significant differences (*p* < 0.05). All experiments in this study were performed with at least three biological replicates.

## 3. Results

### 3.1. Quality Evaluation of BBMVs Protein

The reliability and dependability of the following experiments were determined by the extraction quality of the protein samples. APN and ALP are usually used as marker enzymes in BBMVs from insect midguts [28]. Our results showed that APN and ALP activities in BBMVs samples were approximately 10 times higher than those in the crude enzyme fluid (Table 2). This finding illustrated that APN and ALP are significantly enriched in BBMVs and that the proteins retained their activity after extraction.

### 3.2. Detection and Comparative Analysis of Proteins Separated by 2-DE Gels

**Figure 2:**
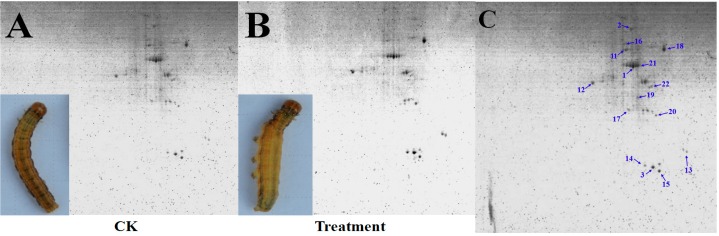

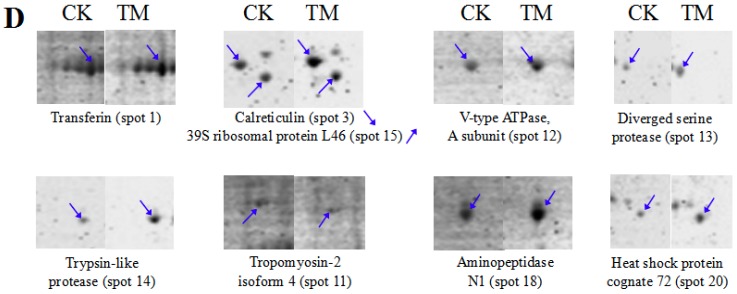
(**A**) Representative spot map of brush border membrane vesicle (BBMVs) from control *M. separata* larvae; (**B**) representative spot map of BBMVs from Periplocoside P (PSP)-treated *M. separata* larvae; (**C**) identification of protein spots through two-dimensional gel electrophoresis (2-DE) and matrix-assisted laser desorption/ionization time of flight/time of flight mass spectroscopy (MALDI-TOF/TOF MS) analysis; and (**D**) magnified views of some differentially expressed proteins (DEPs). CK represents the control *M. separata* group, and TM represents the PSP-treated *M. separata* group. The 2-DE study was conducted with at least three biological replicates.

To understand the response of *M. separata* to PSP, proteins were extracted from the BBMVs of fifth-instar *M. separata* larvae that were either treated with PSP or the control. The proteins were then resolved through 2-DE. The representative spot maps of BBMVs are shown in Figure 2A,B. PDQuest Version 8.0 software and spot-to-spot comparative statistical analysis revealed that PSP-treated larvae exhibited 32 reproducible protein spots, 11 of which had abundance changes (fold change > 1.5). As shown in Figure 2C,D, only one new protein (Spot 2) appeared, and the expression levels of other proteins were either up-regulated or down-regulated. MALDI-TOF/TOF MS (Table 3) results indicated that the identified DEPs included transferrin (Spot 1), protein disulfide isomerase (PDI) precursor (Spot 2), calreticulin (Spot 3), tropomyosin-2 isoform 4 (Spot 11), V-type ATPase (A subunit) (Spot 12), diverged serine protease (Spot 13), trypsin-like protease (Spot 14), 39S ribosomal protein L46 (Mitochondrial-like) (Spot 15), farnesoic acid *O*-methyltransferase (Spot 16), aminopeptidase N1 (Spot 18), and heat-shock protein cognate 72 (HSP72, Spot 20). Tropomyosin-2 isoform 4, 39S ribosomal protein L46 (Mitochondrial-like), and farnesoic acid O-methyltransferase were down-regulated. All other protein spots were all up-regulated. PDI precursor was identified as a newly emerged protein spot.

### 3.3. Functional Analysis of DEPs

**Figure 3:**
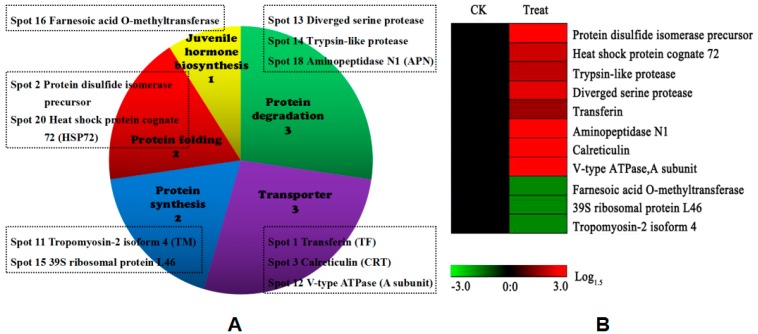
Functional classification (**A**) and hierarchical clustering (**B**) of DEPs. The function of DEPs was elucidated by inputting the gene index with the UniProt accession number in the UniProt database (http://www.uniprot.org). Hierarchical clustering was performed with the log-transformed data using Genesis 1.7.6 software (Graz University of Technology, Austria, http://genome.tugraz. at/genesisclient/genesisclient_download.shtml).

The distributions of DEPs in putative functional categories are shown in Figure 3A. All identified DEPs were assigned to five functional groups, including protein degradation, transporter, protein folding, protein synthesis and juvenile hormone (JH) biosynthesis. Approximately half of DEPs were distributed in protein degradation and transporter. This groups mainly contained transferin, calreticulin (CRT), V-ATPase A subunit, diverged serine protease, and aminopeptidase N1. Protein folding and synthesis had the same proportion of DEPs, including PDI precursor, HSP72 and tropomyosin-2 isoform 4 and 39S ribosomal protein L46. The least-predominant categories contained proteins related to JH biosynthesis. The hierarchical clustering of all DEPs was further analyzed to visualize the protein abundance profiles of all five functional groups (Figure 3B).

**Figure 4:**
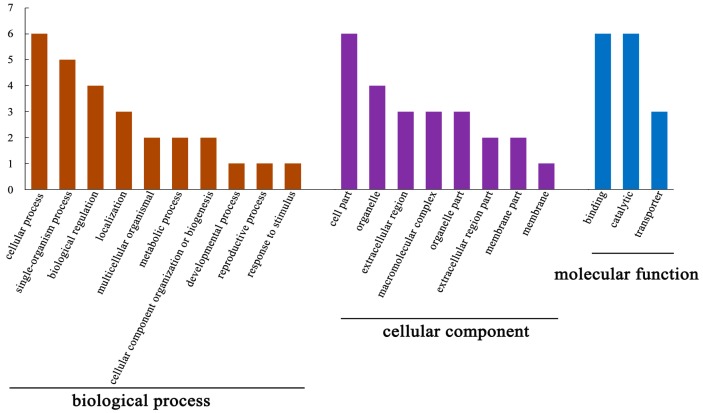
Gene ontology (GO) enrichment analysis of the identified DEPs. GO enrichment was performed with STRING software. BP: cellular process (GO:0015991), single organism process (GO:0006936), biological regulation (GO:0006879), localization (GO:0006826), multicellular organismal process (GO:0007315), metabolic process (GO:0006457), cellular component biogenesis (GO:0045451), developmental process (GO:0045451), reproductive process (GO:0048477), and response to stimulus (GO:0034976). CC: cell part (GO:0005635), organelle (GO:0005783), extracellular region (GO:0005576), macromolecular complex (GO:0033180), organelle part (GO:0005875), extracellular region part (GO:0005615), membrane part (GO:0033180), and membrane (GO:0030017). MF: binding (GO:0005524), catalytic (GO:0004177), and transporter (GO:0046961).

The cellular functions of the DEPs were investigated through GO annotation. The enrichment results for DEPs are shown in Figure 4. Enriched GO annotation revealed that the identified DEPs are mainly implicated in cellular single-organism, multicellular organismal, reproductive, and developmental process, as well as in biological regulation, localization, cellular component biogenesis, and response to stimulus. The DEPs were also categorized in cell, extracellular region, organelle, and membrane parts, as well as macromolecular complex. Moreover, the DEPs were included in three categories related to molecular function: binding, catalytic, and transporter.

### 3.4. Validation of Proteomic Data by RT-qPCR

**Figure 5:**
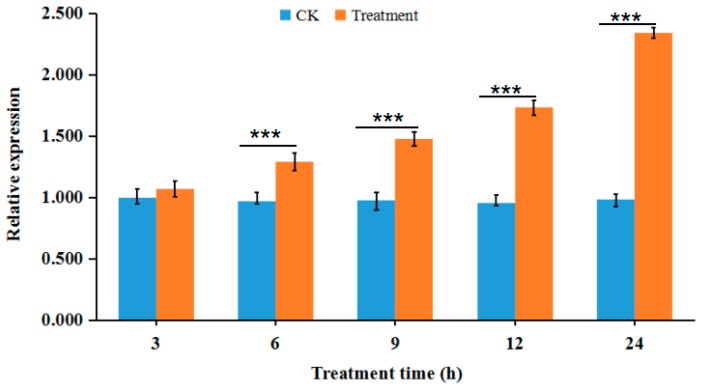
Relative expression levels of *vma1* gene were measured by real-time quantitative polymerase chain reaction (RT-qPCR) at different time-points after PSP treatment. Treatment groups were treated with 4 µmol/L PSP. *β-actin* (GQ856238) was selected as the endogenous control. RT-qPCR analysis was conducted with three biological replicates. Statistical significance was determined through Student’s *t*-test, and significant values were set at *** *p* ≤ 0.001.

The V-ATPase A subunit is encoding by *vma1* and its expression is up-regulated in the oxidative phosphorylation pathway. Meanwhile, enzymology results showed that PSP can significantly suppress V-ATPase activity in a concentration-dependent manner. In this study, we quantified *vma1* expression level through RT-qPCR (Figure 5). At different time-points after treatment, *vma1* expression in the PSP-treated group was higher than that in the control group. Except for that at 3 h after treatment, expression levels were significantly up-regulated at other time-points. The largest fold-change of 2.38 was observed at approximately 24 h after treatment.

### 3.5. Enzymology Verification

**Figure 6:**
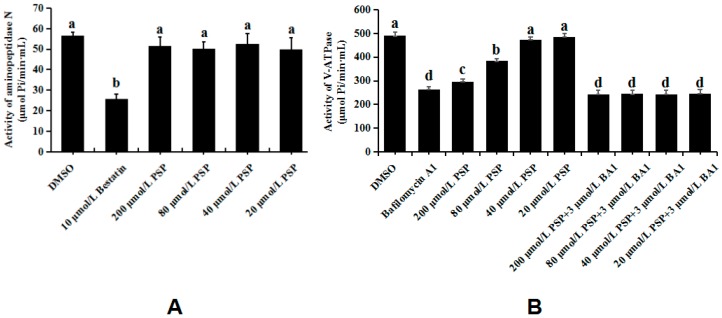
Activities of APN (**A**) and V-ATPase (**B**) under treatment with different concentrations of PSP. Different lowercase letters (a, b, c, and d) indicate significant differences as determined through analysis of variance (*p* < 0.05). This experiment was conducted with five biological replicates.

Given the previous and above results, we speculated that APN and V-ATPase are the initial binding sites of PSP. To verify our hypothesis, we investigated the activities of these two enzymes. As shown in Figure 6A, treatment with PSP or DMSO did not affect APN activity. However, treatment with bestatin, a specific APN inhibitor, significantly suppressed APN activity. Moreover, the activities of V-ATPase were significantly inhibited by treatment with 200 and 80 µmol/L of PSP with inhibition ratios of 39.65% and 21.62%, respectively (Figure 6B). BA1, a macrolide antibiotic known as specific inhibitor of V-ATPase, which can inhibits the V-ATPases specifically without inhibiting other ATPases at concentrations up to 1 µmol/L [29], and binds the membrane-spanning domain of the enzyme to induce a conformational change in the cytosolic domain [30]. It significantly suppressed V-ATPase activity with an inhibition ratio of 46.23% in this study. Meanwhile, we treated larvae with a combination of PSP and BA1 to confirm the specificity of PSP to V-ATPase. The activity of V-ATPase was significantly inhibited by the combination of four different treatments and was not significantly different from that under independent treatment with BA1. If PSP cannot inhibit the V-ATPase activity, the inhibit effect of combined treatment will present summation action, and significantly higher than that of the two treatments alone. These results demonstrated that PSP can significantly suppress V-ATPase activity in a concentration-dependent manner.

## 4. Discussion

In this study, we utilized a proteomics strategy to identify the putative targets of PSP in the midgut BBMVs of *M. separata* larvae. We obtained 32 reproducible protein spots from the BBMVs of *M. separata* larvae through 2-DE, and we successfully identified 11 protein spots through MALDI-TOF/TOF MS. Bioinformatic and functional analysis indicated that these proteins are implicated in the oxidative phosphorylation pathway. RT-qPCR analysis confirmed that the *vma1* gene is highly expressed in midgut BBMVs at different time points after PSP treatment. Finally, enzymology assays further demonstrated that PSP significantly suppresses V-ATPase activity.

Plant secondary metabolites with insecticidal activity are usually small molecule compounds [31] that mainly target enzymes, receptors, and ion channels in insects [32]. In PSP-treated larvae, DEPs with more than two-fold expression changes include CRT, diverged serine protease, trypsin-like protease, APN1, and HSP72. These DEPs participate in physiological and biochemical processes.

CRT and HSP72 induce responses to external stress. CRT, a calcium ion-binding protein that localizes in the endoplasmic reticulum, mainly regulates cellular Ca^2+^ balance and assists in normal protein folding [33]. HSP72, a member of the heat-shock protein family, participate in protein folding and resistance against extracellular stress [34]. HSP72 and CRT can combine with CD91/lRP1 on the surfaces of immune cells to enhance the immune response [35]. In PSP-treated *M. separata* larvae, CRT and HSP72 are up-regulated by immune system response. Trypsin-like protease belongs to the serine protease family. It is responsible for various physiological functions, including protein digestion, protein absorption, and immune response in insects [36]. Its expression was up-regulated by a factor of 2.18. This result is consistent with previous RT-PCR analysis results, and further research indicated that PSP cannot inhibit the activity of purified trypsin-like protease [37]. Therefore, the trypsin-like protease cannot be a target-binding protein of PSP in *M. separata* midguts. The function of PDI, a newly emerged DEP spot, is similar to that of trypsin-like protease and is easily induced by external environmental stress [38]. Therefore, PDI might only be a common phenomenon caused by PSP. APN is another up-regulated DEP. Our previous affinity chromatography study showed that APN is a putative binding proteins of periplocosides [39]. However, enzymological verification revealed that PSP treatment did not significantly affect APN activity.

The V-ATPase A subunit is the most up-regulated DEP under PSP treatment, and its expression level increased by 3.56-folds. RT-qPCR results showed that *vma1* expression is significantly up-regulated at different times-points after PSP treatment. This result is consistent with proteomic results. Further enzymology verification results indicated that V-ATPase can be significantly inhibited by PSP in a concentration-dependent manner. Therefore, V-ATPase, which is implicated in the oxidative phosphorylation pathway, may be associated with the mechanism of action of PSP. V-ATPase, a multi-subunit protein complex, mainly exists in the apical membrane of the goblet cells of the lepidopteran midgut [40,41]. As a specific proton pump, it plays a crucial role in ion transport by regulating intracellular pH equilibration and nutrient uptake by the K^+^/H^+^ antiporter [42]. These functions are dependent on structural changes, such as the dissociation and assembly of V_0_ and V_1_ complexes [43]. The A subunit, a vital subunit of V-ATPase, is positioned in the hydrophilic V_1_ complex that is exposed to the surface of the cell membrane with a globular structure [44]. As the catalytic subunit of V-ATPase, its main function is to catalyze ATP hydrolysis, and generate energy for nutrition secretion and absorption in insects [45]. As for the other subunits of V-ATPase, their expression level did not exhibit significant differences in this study. Therefore, on the basis of bioinformatics analysis and functional characteristics, we speculate that the V-ATPase A subunit in midguts cells may be the initial binding site of PSP in *M. separata* larvae. PSP may first directly bind with the V-ATPase A subunit, thus affecting its catalytic ability to hydrolyze ATP, and eventually causing the overall dysfunction of the midgut system. Alternatively, PSP can directly affect apical membranes in *M. separata* midguts through transmembrane potential depolarization [46] and suppress V-ATPase activity in the malpighian tubules of *Aedes aegypti* [47]. These results are highly consistent with the results in this study, further confirming our hypothesis.

Tropomyosin-2 isoform 4, 39S ribosomal protein L46, and farnesoic acid O-methyltransferase were significantly down-regulated in the BBMVs of PSP-treated *M. separata* larvae. Tropomyosin-2 isoform 4 is an actin-binding protein that regulates the interaction of actin and myosin in the insect muscular system [48]. 39S ribosomal protein L46 participates in protein biosynthesis by forming ribosomes with ribonucleic acid [49]. Farnesoic acid O-methyltransferase is a critical enzyme of JHs, which are key regulators of metamorphosis [50]. Tropomyosin-2 isoform 4, 39S ribosomal protein L46, and farnesoic acid O-methyltransferase have been well studied and are respectively involved in the muscular system, ribosomal formation, and the metamorphosis regulator system. In the present study, their expression levels were significantly down-regulated by approximately 1.93-, 1.91-, and 1.54-folds. However, these effects may have been induced by stress or by the interaction of PSP with its possible initial binding targets. Therefore, we speculate that these three proteins have no direct relationship with the action of PSP.

On the basis of previous electrophysiological research, we suspected that PSP affects the apical membrane potential of *M. separata* midguts by acting on V-ATPase [37]. In this study, we further confirmed this assumption and speculated that the V-ATPase A subunit might be the initial binding site of PSP. We propose a hypothetical mechanism of action for PSP as follows: PSP may affect its catalytic ability to hydrolyze ATP in the oxidative phosphorylation pathway by initially combining with the V-ATPase A subunit. The normal function of V-ATPase in proton transport is then disturbed. This effect leads to the dysregulation of the electrochemical gradient in the midgut, abdominal swelling, and feeding cessation. These effects ultimately lead to insect death.

We hypothesized that the initial binding site of PSP may be the V-ATPase A subunit, with V-ATPase as its main target. However, further validation through site-directed mutagenesis, gene knockout, or RNA interference methods is needed to identify the direct effects of PSP on the V-ATPase A subunit and to identify which amino acid sites of the V-ATPase A subunit are involved. The detection and verification of the putative target and binding site of PSP will help elucidate the specific mechanism of action and development of insecticides based on periplocoside compounds.

## Figures and Tables

**Table 1:** Genes and corresponding primers for real-time quantitative polymerase chain reaction analysis.

Gene	Primer Sequence (5′-3′)
*β-actin*	GGTGTGATGGTTGGTATGGGT
	TCGTTGTAGAAGGTGTGGTGC
*vma1*	TATCCTGGGCTCCATCTTTG
	TTGATGCTCAATGGGTTGAA

**Table 2:** Aminopeptidase N (APN) and alkaline phosphatase (ALP) activities in crude enzyme fluid and brush border membrane vesicle (BBMVs).

Protein Sample	Concentration (mg/mL)	APN Activity (μmol/min·mL)	ALP Activity (μmol/min·mL)
BBMV	0.309	28.169	11.747
Crude enzyme fluid	1.327	3.947	1.829

**Table 3:** Identification of protein spots in PSP-treated *M. separata* larvae compared with those in control *M. separata* larvae.

Spot No.	Protein Name	Accession No.	Matched Peptide Sequences (*m*/*z*)	Score	Relative Molecular Mass (Mr)	Protein Isoelectric Point (p*I*)	Sequence Coverage (%)	Fold Change (*t*-Test *p* < 0.05)
1	Transferin	gi|556559879	HIQALECLR (1139.599)	79	84,436.6	5.79	4	+1.9
			ETAAAQENITR (1203.5964)					
			ASAYTLGIQPAISCQQR (1863.9382)					
2	Protein disulfide isomerase precursor	gi|112984454	NFEEKR (822.4104)	109	55782.2	4.6	11	NA
			QLVPIYDK (975.551)					
			LAEEESPIK (1015.5306)					
			GYPTLKFFR (1128.6201)					
			LIALEQDMAK (1147.6028)					
			LAEEESPIKLAK (1327.7467)					
3	Calreticulin	gi|389608333	AVGEEVKK (859.4883)	50	46,299.2	4.48	2.7	+3.42
			VHVIFSYK (992.5564)					
			KVHVIFSYK (1120.6514)					
			DAGAIAGLNVMR (1491.6533)					
			VESGELEADWDFLPPKK (1959.9698)					
11	Tropomyosin-2 isoform 4	gi|153792609	LIAEESDK (904.4622)	205	29,623	4.77	4.257	–1.93
			EAEARAEFAER (1278.6073)					
			LSEASQAADESER (1392.6238)					
			EEAESEVAALNRR (1473.7292)					
			LSEASQAADESERIR (1661.809)					
			IQLLEEDLERSEER (1758.8868)					
			LLQEEMEATLHDIQNM (1946.8834)					
			TNMEDDRVAILEAQLSQAK (2148.0601)					
12	V-type ATPase, A subunit	gi|71410785	VIVVIPFILLGIP (1392.923)	86	67,985.8	5.6	1.213	+3.56
			MLFVPLGLGQWQLLR (1771.0088)					
			ESGNHPLLTGQR (2002.0215)					
13	Diverged serine protease	gi|2463066	EDMQPLMQDNSD (1438.5461)	98	27,320.9	5.70	0.42	+2.56
			VWLQTCVGSVLTSR (1728.9491)					
14	Trypsin-like protease	gi|2463084	AAVTISSR (804.4574)	107	26,948.1	9.35	5.776	+2.18
			EVPKSEVK (915.5145)					
			ALVSFKIDDK (1135.6357)					
			ELNSLQEKGSK (1232.6481)					
			EVVVKEWYIK (1292.725)					
15	39S ribosomal protein L46, Mitochondrial-like	gi|512919884	SGNPTKSK (818.4366)	135	30,310.5	8.51	2.435	−1.91
			QTAERIVK (944.5523)					
			YKYPSEMNGK (1232.5616)					
			SNHEIQHENDK (1350.6033)					
			IFFYYANYKSGNPTK (1812.8956)					
			LGNDSKTLLPQGHWQEGETLR (2379.2051)					
16	Farnesoic acid O-methyltransferase	gi|528079470	SIPPGALR (810.4832)	30	12,599.4	9.05	1.382	−1.54
			VNHDGCTTPGK (1185.5317)					
			SSAEYECLVLM (1317.5702)					
			KLLSIGDEVNEAVSSMC (1867.8776)					
18	Aminopeptidase N1	gi|34100664	ELAEQEK (846.4203)	154	112,775.9	5.60	6.142	+3.37
			IVEDKNR (873.4788)					
			MNIGNIEAR (1033.5095)					
			FSKCLMDCR (1232.5221)					
			DMLSSLNQEESLK (1493.7152)					
20	Heat shock protein cognate 72	gi|157658	RGGECAR (805.3733)	91	72,234.9	5.22	1.073	+2.33
			YCHRNYGGSK (1241.5481)					
			CPAGLGGNHCEVGRR (1639.754)					
			DIRAEDQTQECNMGDA (1868.7385)					
			FSGISMQVFAIVNGNISPYVLDPNFSHK (3097.5452)					

**Note:** Fold-change: spot abundance was expressed as the ratio of the intensities of up-regulated (plus value) or down-regulated (minus value) proteins between the treatment and control. Fold changes had *p* < 0.05. NA indicates a newly emerged spot. The experiment was conducted with at least three biological replicates.
